# A formation criterion for Order-Disorder (OD) phases of the Long-Period Stacking Order (LPSO)-type in Mg-Al-RE (Rare Earth) Ternary Systems

**DOI:** 10.1038/s41598-017-12506-0

**Published:** 2017-09-25

**Authors:** Kyosuke Kishida, Hideyuki Yokobayashi, Haruyuki Inui

**Affiliations:** 1 Department of Materials Science and Engineering, Kyoto University, Sakyo-ku, Kyoto, 606-8501 Japan; 2 Center for Elements Strategy Initiative for Structural Materials (ESISM), Kyoto University, Sakyo-ku, Kyoto, 606-8501 Japan

## Abstract

The formation of OD (order-disorder) phases of the LPSO (long-period stacking ordered)-type in Mg-Al-RE (RE (rare earth) = Y, La, Ce, Nd, Sm, Dy, Ho, Er and Yb) ternary systems has been investigated for both as-solidified and annealed conditions. The OD phase is found to form in those systems with RE = Y, Nd, Sm, Dy, Ho and Er. The Mg-Al-RE OD phase formed is of the 18*R*-LPSO-type consisting of 6-layer structural blocks with the RE enrichment occurring in the four consecutive atomic layers in the structural block in the form of the Al_6_RE_8_ L1_2_-type atomic clusters. The Mg-Al-RE OD phases are found to be stabilized by the inclusion of any atoms (either Mg, Al or RE) in the central site of the Al_6_RE_8_ L1_2_-type atomic cluster. The occupancy ratio of the central site among Mg, Al and RE atoms varies with the RE element, so that the occupancy ratio of RE atoms increases with the increase in the atomic number of the RE element in particular for the late rare-earth elements. Based on the results obtained, a criterion based on the volume of the Al_6_RE_8_ atomic cluster is proposed to predict the formation of the Mg-Al-RE OD phases.

## Introduction

Mg alloys containing TM (Transition-metal) and RE (Rare-earth) elements have received a considerable amount of attention in recent years as a new-class of light-weight structural materials that can be used in automotive, aerospace and electronics industries in which there is an ever-increasing demand for weight reduction^1–7^. This stems from the fact that these alloys are usually accompanied by plate-shaped precipitate phases with a long-period stacking-ordered (LPSO) structure and exhibit a combination of high strength (>600 MPa) and high ductility (>5% elongation) together with high creep strength after forming by extrusion at high temperatures^1–9^. Although the detailed mechanisms for the achievement of high strength and high ductility for these Mg alloys have not been clear yet, it is generally believed that ternary Mg-TM-RE LPSO phases have played a role^2–11^. In view of the importance of LPSO phases in these Mg alloys, the Mg-TM-RE ternary systems that form LPSO phases have been identified by Kawamura and co-workers^2–5,7^. The RE elements that form LPSO phases in the Mg-Zn-RE systems are classified into two types. Type I includes Y, Dy, Ho and Er, and the LPSO phase is reported to form during solidification in these ternary systems^7^. The LPSO phase formed during solidification is generally based on the 18*R*-type stacking and it transforms into that based on the 14*H*-type stacking upon annealing^4,7,12–14^. On the other hand, type II includes Gd, Tb and Tm, and the LPSO phase of the 14*H*-type is reported to form during annealing while it is absent immediately after solidification^5,8^. Accordingly, they have proposed a criterion for the formation of LPSO phases in the Mg-Zn-RE systems as follows^7^. To form LPSO phases, the RE elements must have (1) negative mixing enthalpy not only with Mg but also with Zn, (2) the hexagonal close-packed (hcp) structure at room temperature, (3) a large solid-solubility (>3.75 at.%) in Mg and (4) an atomic size larger than Mg by 8.4–11.9%^7^.

Recently, LPSO phases have been found in the Mg-Al-Gd system by substituting Zn completely with Al that is not a transition-metal^15–18^. This may indicate that a series of LPSO phases are formed also in Mg-Al-RE ternary systems as in the case of Mg-TM-RE systems. Our recent results on the crystal structure analysis for the 18*R*-type LPSO phase in the Mg-Al-Gd system, however, revealed that the LPSO phase in the Mg-Al-Gd system should not be described as a ‘LPSO’ phase any longer in a strict sense in crystallography because of the existence of the in-plane ordering of Gd and Al atoms in the four consecutive atomic layers enriched with them in the 6-layer structural block^15^. Instead, the crystal structure of the ‘LPSO-type’ phases in the Mg-Al-Gd system is best described with the concept of the order-disorder (OD) structure^15–23^, in which a crystal structure is described with the symmetry of an OD layer (corresponding to a 6-layer structural block) and the relative relation between adjacent two OD layers^15,17,18^. Then, there is a possibility that a series of LPSO-type phases with an OD structure (i.e., OD phases) is formed in Mg-Al-RE ternary systems, although the OD phases have recently been found to develop also in the Mg-Zn-Y system when the Zn/Y concentrations are high^24–26^. It is of importance to note that the in-plane ordering in each structural block of the Mg-Al-Gd OD phases is described as a periodic arrangement of Al_6_Gd_8_ L1_2_-type atomic clusters on lattice points of a two-dimensional $2\sqrt{3}{a}_{{\rm{Mg}}}\times 2\sqrt{3}{a}_{{\rm{Mg}}}$ primitive hexagonal lattice (*a*_Mg_ is referred to the length of the unit vector along the *a*-axis of Mg) (Fig. S1(a) of Supplementary)^15^. In addition, the formation of L1_2_-type atomic clusters is common to both the OD and LPSO phases in the Mg-Al-Gd and Mg-Zn-RE ternary systems^15,16,25,26^ and has been considered to play a key role in the formation of the OD and LPSO phases^17,27–30^. Furthermore, recent first-principles calculations have predicted that the OD phases in the Mg-Al-RE and Mg-TM-RE ternary systems are stabilized by the inclusion of an additional Mg atom in the centre of each L1_2_-type atomic cluster^31–36^. In fact, we have experimentally confirmed that the additional atom inclusion in the OD phases in the Mg-Zn-Y and Mg-Al-Gd systems, although the additional atom is identified not to be restricted to Mg, but is either Mg, Zn or Y and either Mg, Al or Gd^25,26^. Thus, it should be important also to investigate whether the preference of the additional atom included in the L1_2_-type atomic cluster varies depending on the RE element or not for the detailed understanding of the formability of the LPSO-type phases in the Mg-Al-RE and Mg-TM-RE ternary systems.

In the present study, we investigated the formation behaviour of the LPSO-type phases in a number of Mg-Al-RE (RE = Y, La, Ce, Nd, Sm, Dy, Ho, Er and Yb) ternary systems, in order to identify RE elements that form the LPSO-type phases in Mg-Al-RE ternary systems. We also investigated the crystal structures of the LPSO-type phases formed in Mg-Al-RE ternary systems, paying special attention to whether their crystal structures are of the OD-type or not and to whether the preference of the additional inclusion atom in the Al_6_RE_8_ L1_2_-type atomic cluster varies with the RE element or not. Based on the results obtained, a possible empirical criterion for the formation of the LPSO-type phases in Mg-Al-RE ternary systems was discussed.

## Results

### Scanning electron microscopy (SEM) observations

The formation of LPSO-type phases in Mg-Al-RE (RE = Y, La, Ce, Nd, Sm, Dy, Ho, Er and Yb) ternary systems were investigated not only with as-solidified ingots but also with ingots subsequently annealed at either 450 or 500 °C for 64 hours. The results obtained by annealing at 450 °C did not differ significantly from those obtained by annealing at 500 °C. These RE elements are classified into four groups depending on whether or not and how the LPSO-type phase is formed. The identification of LPSO-type phases was made by checking the image intensity, morphology and energy dispersive X-ray spectroscopy (EDS) analysis in the SEM, further confirmed by electron diffraction and imaging in the transmission electron microscopy (TEM) and scanning transmission electron microscopy (STEM). Since LPSO-type phases formed in the Mg-Al-RE systems were indeed all OD phases (as detailed in the subsequent sections), the term, OD phase, will be used hereafter in this paper instead of LPSO phase.

**Figure 1:**
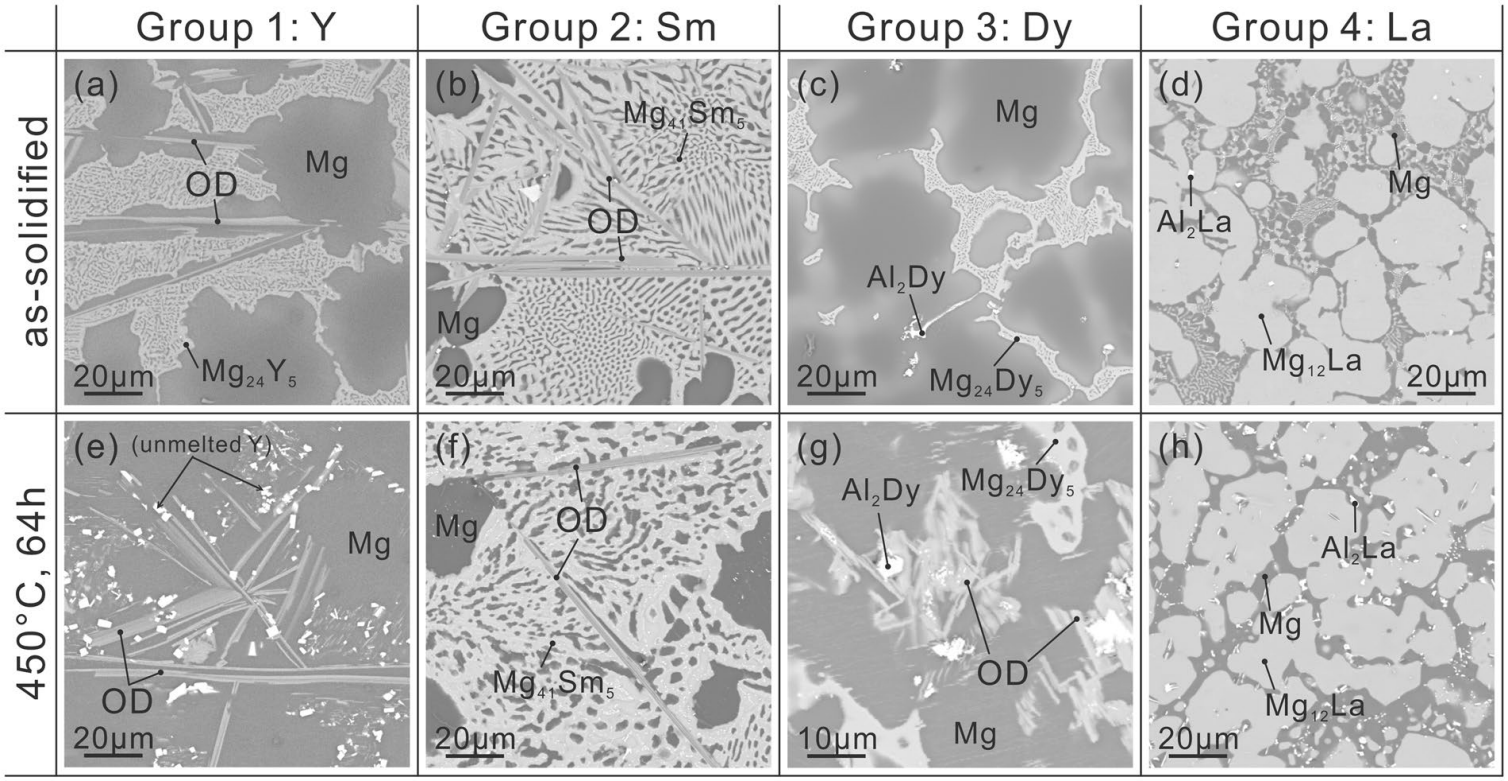
SEM backscattered electron images of (**a**–**d**) the as-solidified ingots and (**e**–**h**) the ingots heat-treated at 450 °C for 64 hours. RE = (**a**,**e**) Y, (**b**,**f**) Sm, (**c**,**g**) Dy and (**d**,**h**) La.

Group 1 consists of Y and Gd^15,17^. In the Mg-Al-RE ternary systems containing these RE elements, the OD phase is observed in the as-solidified ingot and the volume fraction the OD phase increases upon annealing, as shown in Fig. 1(a,e) for the case of RE = Y. Group 2 consists of Nd and Sm. In this case, the OD phase is observed in the as-solidified ingot, and the volume fraction of the OD phase does not change significantly upon annealing at 450 °C, as shown in Fig. 1(b,f) for the case of RE = Sm. However at higher annealing temperature of 500 °C, the OD phase is totally eliminated for both cases of RE = Nd and Sm. Group 3 consists of Dy, Ho and Er. In the Mg-Al-RE ternary systems containing these RE elements, the OD phase is not observed in the as-solidified ingot but it is observed to form upon annealing, as shown in Fig. 1(c,g) for the case of RE = Dy. Group 4 consists of La, Ce and Yb, with which the OD phase is observed neither in the as-solidified ingot nor in the annealed ingot, as shown in Fig. 1(d,h) for the case of RE = La. Of importance to note here is that the OD phases formed in the Mg-Al-RE systems with RE of groups 1~3 coexist with the Mg phase, as the LPSO phases in the Mg-Zn-RE systems do^2–7^.

### Atomic-resolution high angle annular dark-field (HAADF)-STEM observations

**Figure 2:**
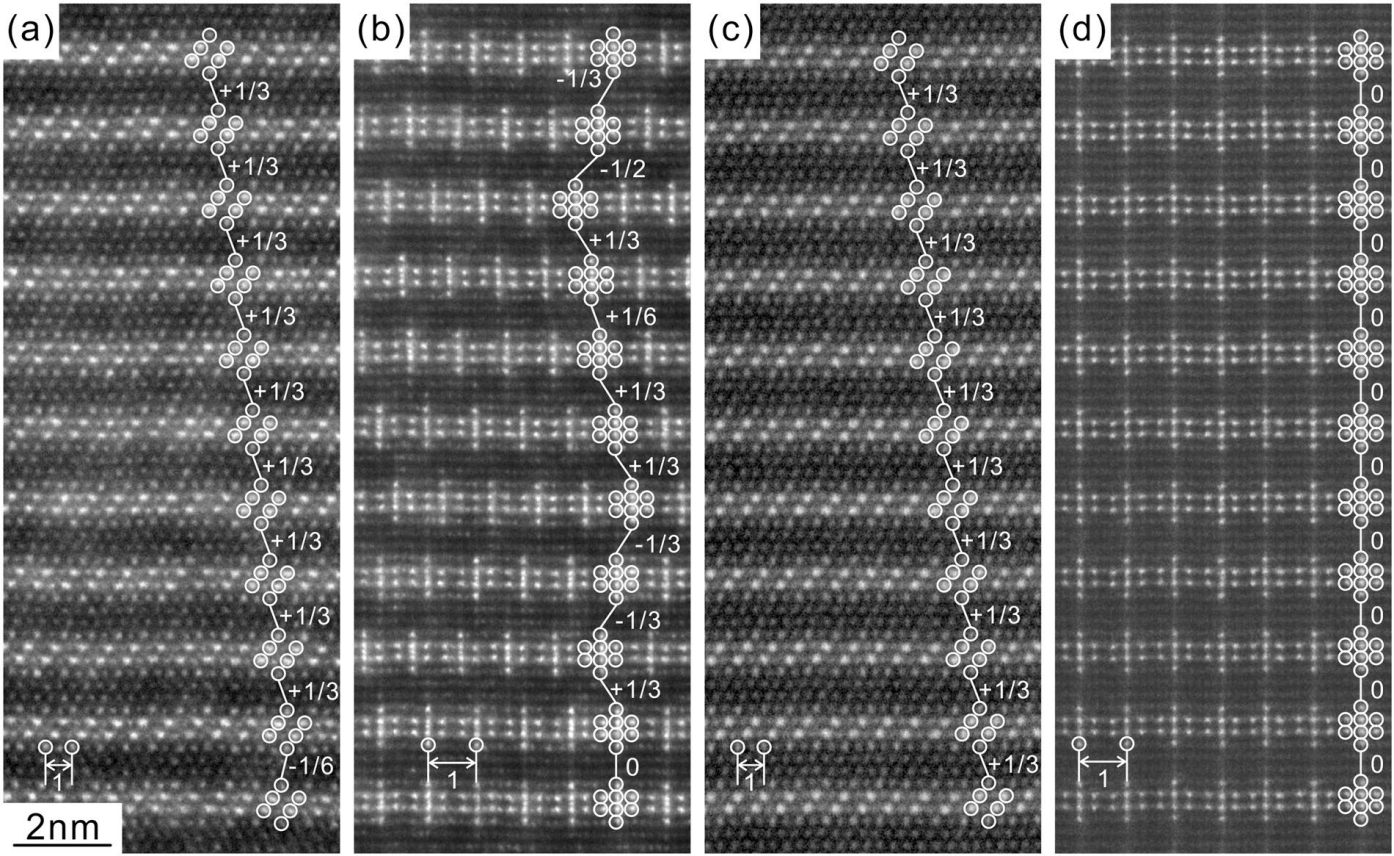
Atomic-resolution HAADF-STEM images of (**a**,**b**) the Mg-Al-Er OD phase in the ingot heat-treated at 450 °C for 64 hours and (**c**,**d**) the Mg-Al-Y OD phase in the ingot heat-treated at 525 °C for 64 hours. The incident beam directions are (**a**,**c**) [$2\bar{1}\bar{1}0$] and (**b**,**d**) [$1\bar{1}00$]. Numbers in the figures indicate the amount of the relative shift between adjacent OD layers in the unit of the projected spacing between adjacent RE-enriched columns in the outer layers of the RE-enriched four consecutive atomic planes (S_1_ and S_2_ in Fig. S1(a) of Supplementary).

Atomic-resolution HAADF-STEM images of the OD phase in the Mg-Al-Er system (group 3) annealed at 450 °C for 64 hours are shown in Fig. 2(a,b) for the incident directions of [2$\bar{1}$ $\bar{1}$0] and [1$\bar{1}$00], respectively. Since the OD phase is known to form in the Mg matrix so that their close-packed directions and planes are parallel to each other, Miller indices to express directions and planes for the OD phase are referred to as those of the matrix phase of Mg with the hcp structure. In these HAADF-STEM images, brighter spots corresponding to atomic columns enriched with RE (Er in this case) with the brightness corresponding to the extent of the enrichment^15,37–40^. The Mg-Al-Er OD phase evidently consists of 6-layer structural blocks of the 18*R*-LPSO-type stacking, within which the enrichment of Er atoms occurs in the central four consecutive atomic layers in the form of Al_6_Er_8_ atomic clusters, as in the case of the OD phase in the Mg-Al-Gd system^15–18^. When judged from the ordered arrangement of ‘double dagger’ patterns corresponding to these Al_6_Er_8_ atomic clusters in the [1$\bar{1}$00] HAADF-STEM image (Fig. 2(b)), the long-range in-plane ordering of Al_6_Er_8_ clusters is completed after annealing at 450 °C for 64 hours. Open circles in Fig. 2(a,b) indicate positions of Er-enriched atomic columns and numbers in the figure indicate the amount of the relative shift occurring between adjacent structural blocks (OD layers) expressed in the unit of the projected spacing between adjacent Er-enriched columns in the outer layers of the four consecutive atomic planes (corresponding to the distance S_1_ and S_2_ in Fig. S1(a) of Supplementary). Inspection of the relative shifts occurring between adjacent structural blocks confirms that the stacking order for 6-layer structural blocks has yet to be completed, as is evident from the sporadic occurrence of the relative shift of 1/2 and 1/6, in addition to the dominant shift of 0 and 1/3. This indicates that stacking positions of C_2_ and C_3_ are also sporadically taken in addition to the dominant C_1_ positions for the stacking of structural blocks in the Mg-Al-Er OD phase at this stage of precipitation. Similar characteristics are observed in the OD phases in the Mg-Al-RE (RE = Y (group 1), Dy and Ho (group 3)) ternary ingots heat-treated at 450 °C for 64 hours. All these are consistent with the result of SAED analysis of Fig. S2 of Supplementary.

The propensity for stacking positions C_2_ and C_3_ is generally decreased upon further annealing so that the OD phase eventually consists of 6-layer structural blocks stacked with only the C_1_ positions. Atomic-resolution HAADF-STEM images of the OD phase in the Mg-Al-Y system annealed at 525 °C for 64 hours are shown in Fig. 2(c,d) for the incident directions [$2\bar{1}\bar{1}0$] and [$1\bar{1}00$], respectively. Inspection of the relative shifts occurring between adjacent structural blocks confirms that the long-range order in the stacking of structural blocks along the [0001] direction is completed in most areas with the occurrence of the regular shift of 1/3 and 0 in the case of Fig. 2(c and d), respectively. This is consistent with the result of SAED analysis that the polytype with the maximum degree of order (MDO polytype), 1 *M* (MDO1, space group: *C*2/*m*) with the simplest stacking in the OD-groupoid family formed with the C_1_ stacking relations is formed as the most stable form for the Mg-Al-Y OD phase, as in the case of the Mg-Al-Gd system^17^.

**Figure 3:**
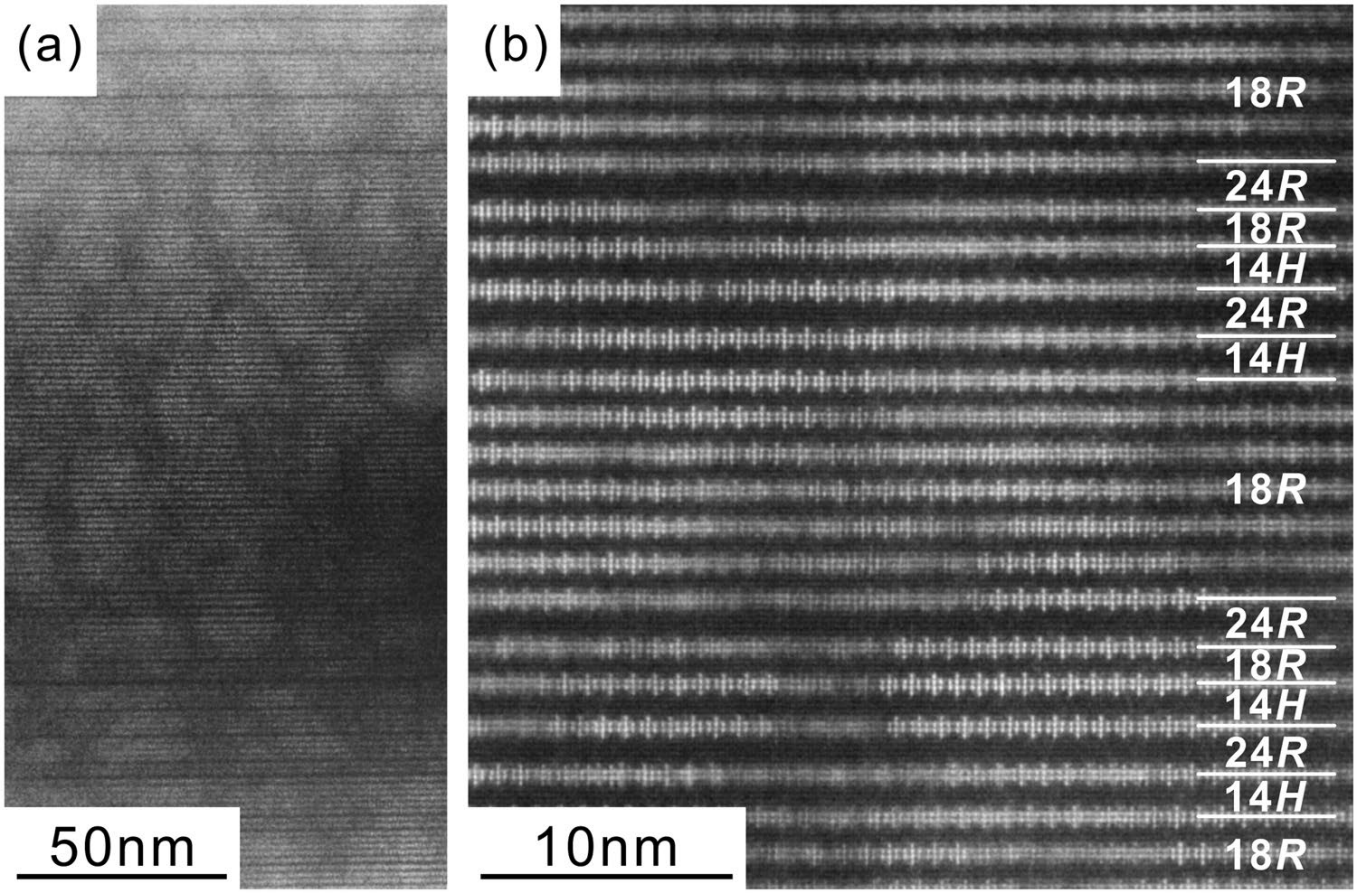
(**a**) Low-magnification and (**b**) atomic-resolution HAADF-STEM images of the Mg-Al-Sm OD phase in the as-solidified ingot. The incident beam direction is [$1\bar{1}00$].

Low-magnification and atomic-resolution HAADF-STEM images of the OD phase in the Mg-Al-Sm system found in the as-solidified ingot are shown in Fig. 3(a and b), respectively. Sm belongs to group 2 with Nd and the OD phase is formed during solidification but eliminated upon annealing at 500 °C for 64 h. The enrichment of Sm occurs in the four consecutive atomic layers in the form of Al_6_Sm_8_ atomic clusters (Fig. 3(b)) even in the as-cast condition. Bright stripes running horizontally in the image of Fig. 3(a) correspond to atomic layers enriched with Sm, and there is obviously no variation in the thickness of bright stripes, indicating the enrichment of Sm occurs always in the form of Al_6_Sm_8_ atomic clusters in four consecutive atomic layers in structural blocks. On the other hand, alternate dark stripes in the image of Fig. 3(a) correspond to Mg layers that sandwich the Sm-enriched four consecutive atomic layers in structural blocks. Although the thickness of these dark stripes is constant in many areas forming 6-layer structural blocks, sporadically occurring thicker dark stripes are observed here and there in Fig. 3(a). These regions with thicker dark stripes correspond locally to structural blocks of either 14*H*- or 24*R*-LPSO-type stacking with 7- and 8- layer structural blocks, as shown in the HAADF-STEM image of Fig. 3(b). In Fig. 3(b), the arrangement of ‘double dagger’ patterns corresponding to Al_6_Sm_8_ atomic clusters is well ordered in some areas but not in other areas, indicating that the formation of Al_6_Sm_8_ atomic clusters already occurs at this stage but that the long-range in-plane ordering of these Al_6_Sm_8_ clusters has not fully completed yet in the as-solidified condition.

### Additional atom at the centre of the Al_6_RE_8_ atomic cluster

**Figure 4:**
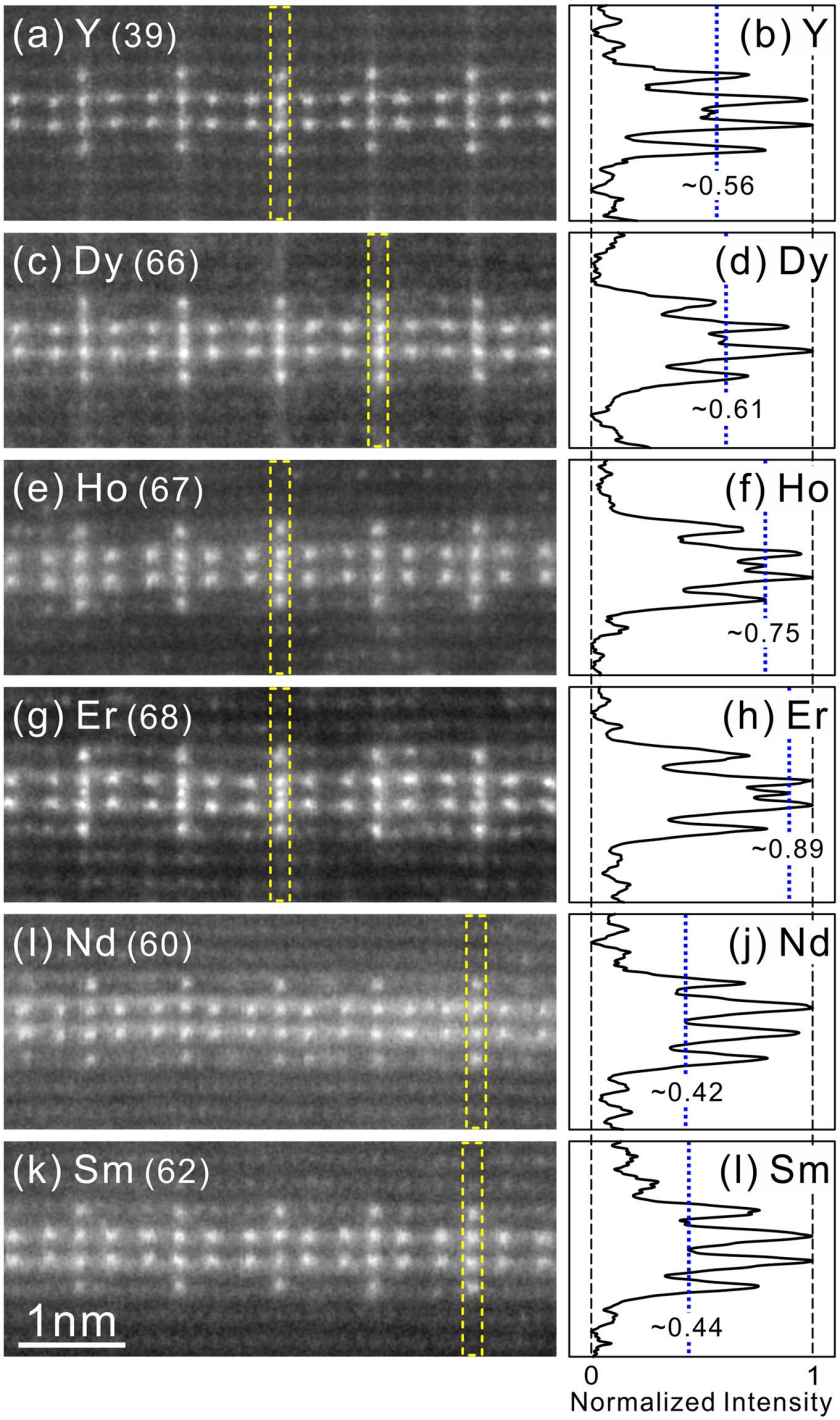
(**a**,**c**,**e**,**g**,**i**,**k**) Ultra-high resolution HAADF-STEM images of various Mg-Al-RE OD phases projected along [$1\bar{1}00$] and (**b**,**d**,**f**,**h**,**j**,**l**) normalized intensity profiles taken along the marked regions in the STEM images. RE = (**a**,**b**) Y, (**c**,**d**) Dy, (**e**,**f**) Ho, (**g**,**h**) Er, (**i**,**j**) Nd and (**k**,**l**) Sm.

The inclusion of an additional atom in the centre of the Al_6_RE_8_ atomic cluster is also observed in the [$1\bar{1}00$] HAADF-STEM images of the OD phases in some Mg-Al-RE systems (Fig. 2). Magnified images of several double-dagger patterns observed in [$1\bar{1}00$] HAADF-STEM images taken from the OD phases in the Mg-Al-RE (RE = Y, Dy, Ho, Er, Nd and Sm) systems are shown in Fig. 4. Additional spots of a relatively high intensity are clearly observed at the position corresponding to the central site of Al_6_RE_8_ atomic clusters for those with RE = Y, Dy, Ho and Er (Fig. 4(a,c,e,g)), while it is less evident for those with RE = Nd and Sm (Fig. 4(i,k)). For each of the OD phases formed with RE = Y, Dy, Ho and Er, the intensity of the additional spots in the centre of Al_6_RE_8_ atomic clusters varies from cluster to cluster, indicating that the element (either Mg, Al or RE) occupying the central position and their occupancies vary from cluster to cluster, as we observed previously for the Mg-Zn-Y OD phases^25,26^. The intensity profile taken from a double dagger pattern including a central additional bright spot with the highest intensity for each of the OD phases (as outlined with dotted lines in Fig. 4(a,c,e,g,i,k)) are shown in Fig. 4(b,d,f,h,j,l). Normalization of these intensity profiles is made by taking the highest and lowest intensities in the image profile unity and zero; the highest intensity is exhibited by the brightest spot corresponding to the RE atom column in either of the central two atomic layers of the RE enrichment while the lowest intensity is exhibited by the background between adjacent pure Mg layers. The normalized intensity at the position of the additional spot is found to increase in the order of Y, Dy, Ho and Er, i.e. in the increasing order of the atomic number. The fraction of double-dagger patterns accompanied by an additional bright spot with a relatively high intensity seems to be larger in the Mg-Al-Er OD phase (Figs 2(b) and 4(d)) than those in the Mg-Al-Y (Figs 2(d) and 4(a)), Mg-Al-Dy (Fig. 4(b)) and Mg-Al-Ho (Fig. 4(c)) OD phases. In view of the fact that the intensity of bright spots (corresponding to atomic columns) in atomic resolution HAADF-STEM image is approximately proportional to the square of the average atomic number of the atomic column^37–40^, these observations suggest that the occupancy ratio of the central site among Mg, Al and RE atoms varies with the RE element, so that the occupancy ratio by RE atoms increases with the increase in the atomic number of the RE element. For light rare-earth elements, Nd and Sm, although no apparent additional spot is observed in the centre of each double dagger pattern, the intensity at the additional spot position is far above that calculated assuming without any additional atom at the central position. This may indicate that the central sites of Al_6_RE_8_ atomic clusters in the OD phases formed with RE = Nd and Sm are occupied more by Mg (or Al) atoms than by the RE atoms.

### First-principles calculations for the OD phases in the Mg-Al-RE systems

**Figure 5:**
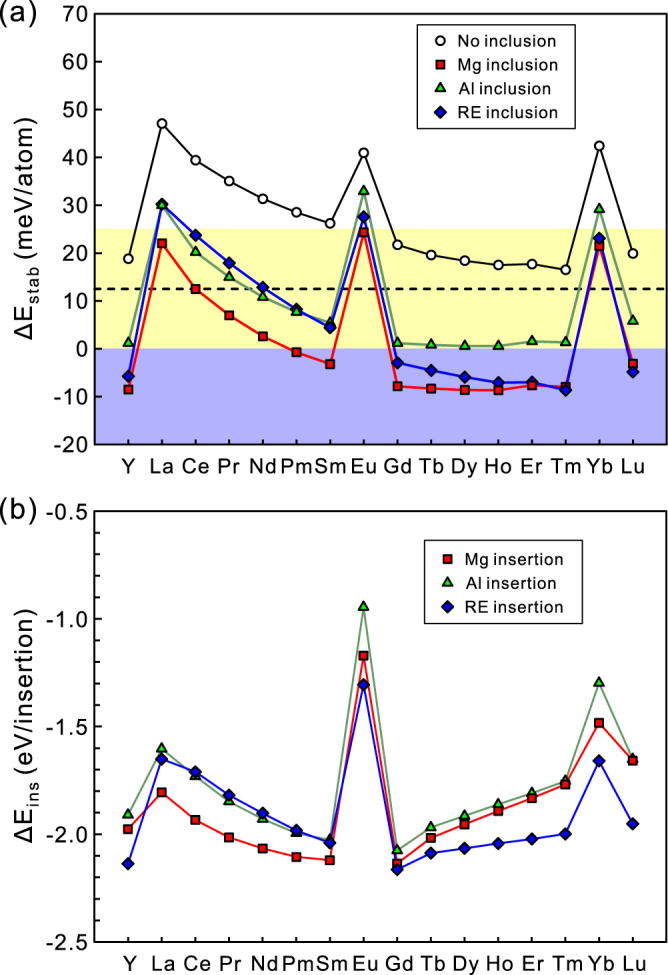
(**a**) Stability factor ΔE_stab_ and (**b**) insertion energy ΔE_ins_ for the 18*R*-LPSO-type Mg-Al-RE OD phase. Blue and yellow bands in (**a**) indicate the regions with ΔE_stab_ < 0 (OD phase is stable) and ΔE_stab_ < 25 meV/atom (OD phase is nearly stable) according to the stability criteria proposed by Saal and Wolverton^35^, respectively. A Broken line indicates the approximate upper limit of ΔE_stab_ for the OD phase formation deduced based on the experimental results.

In order to deduce the possible reasons for the RE inclusion at the central site of Al_6_RE_8_ atomic clusters, first-principles density functional theory (DFT) calculations were conducted using the Vienna Ab initio simulation package (VASP)^41^. The OD-phase formation energy ΔE_form_, stability factor ΔE_stab_ and energy required to insert one additional atom *i* (*i* = Mg, Al or RE) in each Al_6_RE_8_ atomic cluster ΔE_ins_ (insertion energy) were evaluated with the following equations^25,26,34,35^:

$${{\rm{\Delta }}{\rm{E}}}_{{\rm{f}}{\rm{o}}{\rm{r}}{\rm{m}}}={{\rm{E}}}_{{\rm{t}}{\rm{o}}{\rm{t}}}({{\rm{M}}{\rm{g}}}_{l}{{\rm{A}}{\rm{l}}}_{m}{{\rm{R}}{\rm{E}}}_{n})-\{l{{\rm{E}}}_{{\rm{t}}{\rm{o}}{\rm{t}}}({\rm{M}}{\rm{g}})+m{{\rm{E}}}_{{\rm{t}}{\rm{o}}{\rm{t}}}({\rm{A}}{\rm{l}})+n{{\rm{E}}}_{{\rm{t}}{\rm{o}}{\rm{t}}}({\rm{R}}{\rm{E}})\}/(l+m+n)$$

$${{\rm{\Delta }}{\rm{E}}}_{{\rm{s}}{\rm{t}}{\rm{a}}{\rm{b}}}({{\rm{M}}{\rm{g}}}_{l}{{\rm{A}}{\rm{l}}}_{m}{{\rm{R}}{\rm{E}}}_{n})={{\rm{E}}}_{{\rm{t}}{\rm{o}}{\rm{t}}}({{\rm{M}}{\rm{g}}}_{l}{{\rm{A}}{\rm{l}}}_{m}{{\rm{R}}{\rm{E}}}_{n})-{{\rm{E}}}_{{\rm{C}}{\rm{H}}}({{\rm{M}}{\rm{g}}}_{l}{{\rm{A}}{\rm{l}}}_{m}{{\rm{R}}{\rm{E}}}_{n})$$

$${{\rm{\Delta }}{\rm{E}}}_{{\rm{i}}{\rm{n}}{\rm{s}}}(i)={{\rm{E}}}_{{\rm{t}}{\rm{o}}{\rm{t}}}({{\rm{M}}{\rm{g}}}_{l}{{\rm{A}}{\rm{l}}}_{m}{{\rm{R}}{\rm{E}}}_{n}+i)-{{\rm{E}}}_{{\rm{t}}{\rm{o}}{\rm{t}}}({{\rm{M}}{\rm{g}}}_{l}{{\rm{A}}{\rm{l}}}_{m}{{\rm{R}}{\rm{E}}}_{n})-{{\rm{E}}}_{{\rm{t}}{\rm{o}}{\rm{t}}}(i)$$

where E_tot_(*j*) is the total energy per atom of the phase *j* and E_CH_(Mg_*l*_Al_*m*_RE_*n*_) is the energy of the convex hull at the composition of the OD phase with a formula unit of Mg_*l*_Al_*m*_RE_*n*_ ((*l*, *m*, *n*) = (58, 6, 8) when no additional atom is inserted). Each convex hull is composed of three convex hull phases mostly selected according to the selections made by Saal and Wolverton except for the Mg-Al-Pm ternary system^35^ (listed in Table S1 of Supplementary). According to the stability criteria proposed by Saal and Wolverton^35^ that the OD phase is considered to be stable and nearly stable respectively if ΔE_stab_ < 0 and ΔE_stab_ < 25 meV/atom, all of the 18*R*-LPSO-type Mg-Al-RE OD phases (RE = Y, La ~ Lu) are predicted to be stabilized when an Mg atom is inserted at the centre of each Al_6_RE_8_ atomic cluster (Fig. 5(a), Table S1 of Supplementary). This is fully consistent with the previous reports^34,35^. It should be noted, however, that the difference in the stability factor ΔE_stab_ for the Mg inclusion and the RE inclusion decreases with the increase in atomic number for the heavy rare-earth elements (RE = Gd ~ Tm), while it does not change significantly for the light rare earth-elements (RE = La ~ Sm). The results of calculation of the stability factors ΔE_stab_ (Fig. 5(a)) and the insertion energy ΔE_ins_ (Fig. 5(b)) indicate that the RE inclusion in the central site of the Al_6_RE_8_ atomic cluster is increasingly more favourable for the Mg-TM-RE OD phases with the heavier rare-earth elements (RE = Gd ~ Tm). This may be the reason for the present STEM observations that the fraction of double-dagger patterns containing an additional bright spots with relatively high intensity increases with the increase in atomic number of RE atoms that form the Mg-Al-RE OD phases (Fig. 4). Based on the comparison between the experimental and calculation results, the stability criteria of the Mg-Al-RE OD phases would be described approximately as follows: the OD phase is expected to be stable if ΔE_stab_ < 12.5 meV/atom, the value of which corresponds to that for the Mg-Al-Ce OD phase with the Mg inclusion.

## Discussion

In the present study, some rare-earth elements (RE = Y, Nd, Sm, Dy, Ho and Er) have been found to form the Mg-Al-RE OD phases in addition to Gd, while others (RE = La, Ce and Yb) not. We first discuss the applicability of the criterion propose by Kawamura and co-workers for the formation of the LPSO phases in the Mg-Zn-RE ternary systems^7^ to the formation of the OD phases in the Mg-Al-RE systems. Table 1 summarizes the mixing enthalpy of Mg-RE, Al-RE pairs, maximum solubility in Mg and metallic radius for various RE atoms^42,43^ that are required to check the applicability of the proposed criterion as described in the Introduction section. The rare-earth elements (RE = Y, Nd, Sm, Gd, Dy, Ho and Er) that form the Mg-Al-RE OD phase all satisfy the criterion items (1) and (4) related respectively to mixing enthalpies and metallic radius. However, the items (2) related to crystal structure and (3) related to solubility limit are violated by Nd (dhcp structure, maximum solubility ~0.63 at. %) and Sm (rhombohedral, maximum solubility ~0.99 at. %). This indicates that the criterion proposed for the LPSO-phase formation in the Mg-Zn-RE ternary systems is not simply applicable to the OD-phase formation in the Mg-Al-RE systems.

**Table 1:** Mixing enthalpy of Mg-RE and Al-RE pairs, maximum solubility of RE in Mg, metallic radii, crystal structures at room temperature and average atomic volume for Mg, Al and RE elements^42–44^.

	Mixing enthalpy (kJ/mol)		Maximum solubility in Mg (at.%)	Metallic radius (Å)	Crystal structure at room temperature	Average atomic volume (Å^3^)
	Mg	Al				
Mg	—	−2	—	1.602	hcp	23.09
Al	−2	—	11.8	1.432	fcc	16.61
Y	−6	−38	3.59	1.8012	hcp	33.01
La	−7	−38	0.042	1.8791	dhcp	37.42
Ce	−7	−38	0.13	1.8247	fcc	34.78
Pr	−6	−38	0.31	1.8279	dhcp	34.53
Nd	−6	−38	0.63	1.8214	dhcp	34.17
Pm	−6	−39	0.78	1.811	dhcp	33.60
Sm	−6	−38	0.99	1.8041	rhombohedral	33.18
Eu	−5	−19	4.8 × 10^−5^	2.0418	bcc	48.13
Gd	−6	−39	4.48	1.8013	hcp	33.00
Tb	−6	−39	4.57	1.7833	hcp	32.13
Dy	−6	−38	4.83	1.774	hcp	31.60
Ho	−6	−38	5.44	1.7661	hcp	30.82
Er	−5	−38	6.59	1.7566	hcp	30.56
Tm	−5	−38	6.29	1.7462	hcp	30.29
Yb	−6	−20	0.48	1.9392	fcc	41.25
Lu	−5	−39	8.8	1.7349	hcp	29.90

**Figure 6:**
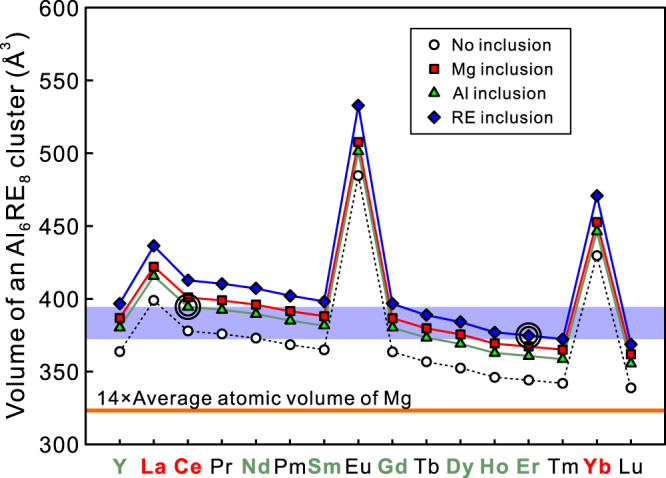
Volume of an Al_6_RE_8_ atomic cluster with and without inclusion of an additional atom at the central site of each Al_6_RE_8_ atomic cluster. A pale blue band indicates the possible size range for the successful formation of the Mg-Al-RE OD phases.

We now discuss an alternative criterion for the formation of the OD phases in the Mg-Al-RE systems. Since the OD phase is characterized by a very high degree of in-plane ordering of dense Al_6_RE_8_ atomic clusters with a periodic arrangement on lattice points of a two-dimensional $2\sqrt{3}{a}_{{\rm{Mg}}}\times 2\sqrt{3}{a}_{{\rm{Mg}}}$ primitive hexagonal lattice, the formability of the OD phase is considered to be closely related to the stability of Al_6_RE_8_ atomic clusters in the OD phase as well as the inter-cluster interaction among them^27–30^. Because both the LPSO and OD structures can be described approximately as the structures containing dispersed Al_6_RE_8_ atomic clusters embedded in the Mg matrix^27,29^, the size and number density of the Al_6_RE_8_ atomic clusters are important factors controlling the stability of the OD phase. Here, we propose an alternative criterion for the formation of the OD phases in the Mg-Al-RE systems based on the volume of the Al_6_RE_8_ atomic cluster. This partly comes from the fact that the in-plane density of Al_6_RE_8_ atomic clusters is virtually the same (with the two-dimensional $2\sqrt{3}{a}_{{\rm{Mg}}}\times 2\sqrt{3}{a}_{{\rm{Mg}}}$ primitive hexagonal lattice) for all observed Mg-Al-RE OD phases and also comes from the fact that the average volume of the Al_6_RE_8_ atomic cluster is one of the simplest factors altered by the type and amount of the inclusion atoms in the Al_6_RE_8_ atomic clusters. Figure 6 shows the variation of the volume of the Al_6_RE_8_ atomic cluster with an additional atom. The cluster volumes, *V*_cluster_, are estimated with the average atomic volume deduced for pure elements, ${\bar{V}}_{{\rm{Mg}}}$, ${\bar{V}}_{{\rm{Al}}}$ and ${\bar{V}}_{{\rm{RE}}}$ (unit cell volume divided by the number of atoms in the unit cell) as follows,

4, $${V}_{{\rm{c}}{\rm{l}}{\rm{u}}{\rm{s}}{\rm{t}}{\rm{e}}{\rm{r}}}=6{\bar{V}}_{{\rm{A}}{\rm{l}}}+8{\bar{V}}_{{\rm{R}}{\rm{E}}}+{\bar{V}}_{{\rm{i}}{\rm{n}}{\rm{c}}{\rm{l}}}$$

where ${\bar{V}}_{{\rm{incl}}}$ is either 0, ${\bar{V}}_{{\rm{Mg}}}$, ${\bar{V}}_{{\rm{Al}}}$ or ${\bar{V}}_{{\rm{RE}}}$ depending on the central site of the Al_6_RE_8_ atomic cluster is not occupied or is occupied by either Mg, Al or RE. The values of the average atomic volumes for pure elements estimated using the crystallographic information in ref.^44^ are summarized in Table 2. It is now obvious from previous theoretical and experimental investigations^25,26,31–36^ as well as from the present theoretical calculation that the central site of the Al_6_RE_8_ atomic cluster is occupied by either Mg, Al (or TM) or RE. Thus, it is quite reasonable to assume that the Mg-Al-RE OD phase formation should always be accompanied by the atom inclusion in the Al_6_RE_8_ atomic cluster whatever the atom kind is. Then, for any particular Mg-Al-RE OD phase, the volume of the Al_6_RE_8_ atomic cluster is the smallest with the Al inclusion and is the largest with the RE inclusion (Fig. 6). On the assumption, we can determine the cluster volume range for the successful formation of the OD phase as indicated with a pale blue band in Fig. 6. The upper limit is determined by the volume of Al_6_Ce_8_ atomic cluster with the Al inclusion, since the OD phase is not formed in the Mg-Al-Ce systems. The lower limit of the range is tentatively determined by the volume of Al_6_Er_8_ atomic cluster with the Er inclusion, since the cluster volume in the Mg-Al-Er OD phase is the smallest among all OD phases confirmed to form. If the OD phase formation is confirmed in the Mg-Al-Tm and Mg-Al-Lu systems, the lower limit should be further lowered accordingly. The deduced volume range for the formation of the Mg-Al-RE OD phases is 374.7 ≤ *V*_cluster_ < 394.5 (Å^3^), which is 15.9 ~ 22.0% larger than the corresponding volume of Mg (14 ${\bar{V}}_{{\rm{Mg}}}$ = 323.3 (Å^3^)). In other words, the OD phases are predicted to form in the Mg-Al-RE systems if the average atomic volume of RE atoms ${\bar{V}}_{{\rm{RE}}}$ is in the range of 30.56 ≤ ${\bar{V}}_{{\rm{RE}}}$ < 34.78 (Å^3^). According to this criterion, the OD phase is expected to form in the Mg-Al-RE ternary systems with RE = Pr, Pm, and Tb as well. This has yet to be investigated.

Figure 6 also predicts that while the possibility for the Al_6_RE_8_ atomic cluster to have the RE inclusion in the central site is quite low for the early rare-earth atoms (La~Sm), the possibilities for the RE and Mg inclusions respectively increase and decrease with the increase in atomic number of RE atoms for the late rare-earth atoms (Gd~Er). This is fully consistent with the atomic-resolution HAADF-STEM observations of Fig. 4. This indicates that occupancies of either Mg, Al or RE in the central site of the Al_6_RE_8_ atomic cluster are also determined by the volume range of the Al_6_RE_8_ atomic cluster for the successful formation of the Mg-Al-RE OD phases.

## Conclusions

We investigated the formation of OD phases of the LPSO-type in Mg-Al-RE (RE = Y, La, Ce, Nd, Sm, Dy, Ho, Er and Yb) ternary systems by SEM, TEM and atomic resolution STEM. The results obtained are summarized as follows.

1. The RE elements are classified into four groups, depending on whether or not and how the OD phase is formed. Group 1 consists of Y and Gd, and the OD phase forms even during solidification and the volume fraction of the OD phase increases upon annealing. Group 2 consists of Nd and Sm, and the volume fraction of the OD phase formed during solidification does not significantly change upon annealing. Group 3 consists of Dy, Ho and Er, and the OD phase does not form during solidification but does upon annealing. Group 4 consists of La, Ce and Yb, with which the OD phase is observed neither in the as-solidified ingot nor in the annealed ingot.
2. The Mg-Al-RE OD phase formed with the RE elements belonging to groups 1–3 consists of 6-layer structural blocks stacked on top of each other by taking preferentially the C_1_ stacking relations among the possible three crystallographically different relations. The enrichment of RE atoms occurs in the four consecutive atomic layers in the structural block in the form of the Al_6_RE_8_ L1_2_-type atomic clusters. The in-plane ordering of the Al_6_RE_8_ L1_2_-type atomic clusters occurs from the early stage of precipitation accompanied by a periodic arrangement of Al_6_RE_8_ atomic clusters on lattice points of a two-dimensional $2\sqrt{3}{a}_{{\rm{Mg}}}\times 2\sqrt{3}{a}_{{\rm{Mg}}}$ primitive hexagonal lattice.
3. The most stable polytype of the Mg-Al-Y OD phase is confirmed to be the simplest MDO polytype, 1 *M* (MDO1) with the space group of *C*2/*m* in the OD-groupoid family formed with the C_1_ stacking relations, as in the case of the Mg-Al-Gd OD phase^17^.
4. The OD phases in the Mg-Al-RE systems are found to be stabilized by the inclusion of any atoms (either Mg, Al or RE) in the central site of the Al_6_RE_8_ atomic cluster. The occupancy ratio of the central site among Mg, Al and RE atoms varies with RE element with which the OD phase is formed, so that the occupancy ratio of RE atom increases with the increase in the atomic number of RE elements in particular for the late rare-earth elements.
5. We propose a criterion for the formation of the OD phases in the Mg-Al-RE systems based on the volume of the Al_6_RE_8_ atomic cluster. The criterion states that the Mg-Al-RE OD phases is formed if the volume of the corresponding Al_6_RE_8_ atomic cluster is in the range of 374.7 ≤ *V*_cluster_ < 394.5 (Å^3^), which is 15.9 ~ 22.0% larger than the corresponding volume of Mg (14 ${\bar{V}}_{{\rm{Mg}}}$ = 323.3 (Å^3^)). Alternatively, the OD phases are predicted to form in the Mg-Al-RE systems if the average atomic volume of RE atoms is in the range of is 30.56 ≤ ${\bar{V}}_{{\rm{RE}}}$ < 34.78 (Å^3^).

## Methods

Ingots of Mg-Al-RE (RE = Y, La, Ce, Nd, Sm, Dy, Ho, Er and Yb) ternary alloys were produced by high-frequency induction-melting mixtures of high-purity Mg, Al and RE amounting to a nominal composition of Mg − 3.5 at. % Al − 7.0 at. % RE in a carbon crucible in vacuum. After melting, the ingots were quickly removed from the furnace to observe the as-solidified microstructures. Then, the ingots were annealed at either 450, 500 or 525 °C for 64 hours followed by water-quenching. Microstructures were examined by SEM with a JEOL JSM-7001FA electron microscope, TEM and STEM with JEOL JEM-2000FX and JEM-ARM200F electron microscopes. Chemical compositions were analysed by EDS in the SEM. Specimens for SEM, TEM observations were cut from as-solidified and annealed ingots, mechanically polished and electropolished in a solution of nitric acid and methanol (9:21 by volume) under a constant voltage of 12–22 V at −55 °C. Specimens for high-resolution STEM observations were prepared by Ar-ion milling method using a JEOL EM-9100IS ion milling machine.

First-principles DFT calculations were conducted using the VASP code^41^. The generalized gradient approximation of Perdew-Burke-Ernzerhof (GGA-PBE) is used to treat the exchange-correlation functional^45^. The crystal structure used for the calculations is the MDO polytype, 1 *M* (MDO1, space group: *C*2/*m*) in the OD-groupoid family formed with the C_1_ stacking relations as observed in the Mg-Al-Y and Mg-Al-Gd systems^17^. Four model cells with and without an additional atom of either Mg, Al or RE at the central site of the Al_6_RE_8_ atomic cluster are calculated. An energy cutoff is set to be 400 eV and Monkhorst-Pack *k*-point mesh of 6 × 4 × 4 is used throughout the calculations^46^. The geometric optimization is terminated when the residual forces become less than 0.01 eV/Å.

### Data availability

The datasets generated during and/or analysed during the current study are available from the corresponding author on reasonable request.

## Electronic supplementary material

Supplementary Information
