# Supplementary material for: A formation criterion for Order-Disorder (OD) phases of the Long-Period Stacking Order (LPSO)-type in Mg-Al-RE (Rare Earth) Ternary Systems
Source: Sci Rep. 2017 Sep 25;7:12294. doi: 10.1038/s41598-017-12506-0 (PMC5612944; doi:10.1038/s41598-017-12506-0)
Supplement: Supplementary file 1 — Supplementary Information [file 41598_2017_12506_MOESM1_ESM.pdf]

Supplementary Information

A formation criterion for Order-Disorder (OD) phases of the Long-Period Stacking Order (LPSO)-type in Mg-Al-RE (Rear Earth) Ternary Systems

Kyosuke Kishida[1,2,*], Hideyuki Yokobayashi[1], and Haruyuki Inui[1,2]

[1]Department of Materials Science and Engineering, Kyoto University, Sakyo-ku, Kyoto, 606-8501 Japan
[2]Center for Elements Strategy Initiative for Structural Materials (ESISM), Kyoto University, Sakyo-ku, Kyoto 606-8501, Japan
*kishida.kyosuke.6w@kyoto-u.ac.jp

S1. The OD structure of the 18*R*-LPSO-type phase

The 18*R*-LPSO-type phase in the Mg-Al-Gd system is crystallographically described as an OD phase formed by stacking structural blocks, each of which consists of six close-packed atomic layers[1,2]. The in-plane long-range ordering of Gd and Al in the central four consecutive atomic layers in each structural block can be described as a periodic arrangement Al_6Gd_8 clusters with the $L1_2$-type atomic arrangement on lattice points of a two-dimensional $2\sqrt{3}a_{\mathrm{Mg}} \times 2\sqrt{3}a_{\mathrm{Mg}}$ primitive hexagonal lattice, where a_{Mg} is referred to the length of the unit vector along the *a*-axis of Mg (Fig. S1(a)). For the Mg-Al-Gd OD phase, the 6-layer structural block corresponds to the OD layer with the layer group symmetry of $P(\bar{3})1m$[1-4]. Three different OD-groupoid families can be derived depending on stacking relations between neighboring OD layers and the layer group symmetry of the OD layer[1,2]. These three OD-groupoid families are characterized with three different types of stacking positions (C_1-C_3 in Fig. S1(b))[1,2]. Among the three possible OD-groupoid families, the OD phase in the Mg-Al-Gd system belongs to the OD-groupoid family characterized with the C_1 stacking positions. There are three crystallographically equivalent stacking relations of the C_1-type as described with the following stacking vectors (Fig. S1(b)),

$$\mathbf{t}_1 = -\frac{1}{3}\mathbf{a}_1 + \mathbf{h},\ \mathbf{t}_2 = -\frac{1}{3}\mathbf{a}_2 + \mathbf{h}, \text{ and } \mathbf{t}_3 = \frac{1}{3}(\mathbf{a}_1 + \mathbf{a}_2) + \mathbf{h} \qquad \text{(S1)},$$

where $\mathbf{a}_1$ and $\mathbf{a}_2$ correspond to the unit vectors of the structural block in the hexagonal setting and $\mathbf{h}$ corresponds to the unit vector of the 6-layer structural block along the stacking direction perpendicular to $\mathbf{a}_1$ and $\mathbf{a}_2$. For the single OD-groupoid family, many different polytypes with a wide range of periodicity along the stacking direction can be deduced by assuming different combinations of stacking vectors described in eq. (1). In any OD-groupoid family, there exist some structurally simple polytypes called polytypes with the maximum degree of order (MDO polytypes)[2,5-7]. The simplest MDO polytypes, 1*M* (MDO1, space group: *C*2/*m*) in the Ramsdell notation was confirmed to be the most stable form of the OD phase in the Mg-Al-Gd system[8].

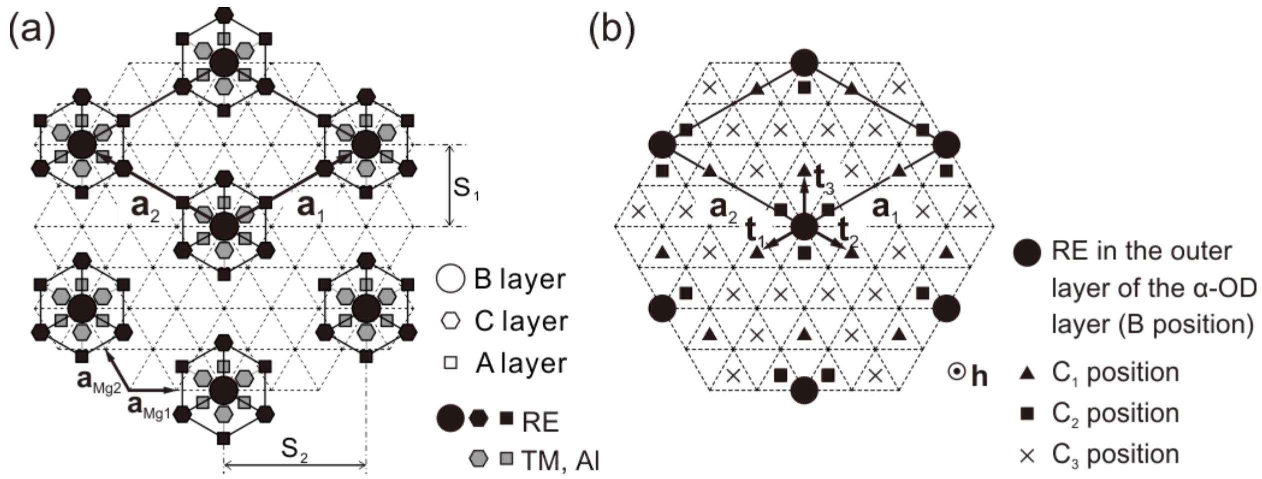

Figure S1. (a) In-plane ordering of Al_6RE_8 $L1_2$ atomic clusters in the OD layer of the Mg-Al-RE OD phase projected along [0001]. (b) Possible stacking positions of the β-OD layer (stacking sequence: BCBACA) stacked on top of the α-OD layer (stacking sequence: ABACBC) for the 18*R*-LPSO-type Mg-Al-RE OD phase. Positions of the α- and β-OD layers are indicated with those of the RE atoms in the outer RE-enriched atomic layers of the four consecutive RE enriched atomic layers in the B and C positions, respectively.

S2. Electron diffraction

Structural characteristics of OD structures, such as OD-groupoid families (preferential stacking positions for the OD layers) and polytypes (stacking sequences of the OD layers), can be extracted from their electron diffraction patterns. For crystals belonging to an OD-groupoid family, reflections are classified into two types; OD 'family' reflections common to all possible polytypes belonging to the OD-groupoid family, and 'characteristic' reflections depending on polytypes [1,2,5,8,9]. The identification of the OD-groupoid family can be made by inspecting positions of family reflections, while that of polytype can be determined from those of characteristic reflections. If the three crystallographically equivalent C_1 stacking relations (equation (S1)) occur randomly in stacking OD layers, the OD phase exhibits a one-dimensionally disordered nature. Then, the reciprocal lattice rows of the characteristic reflections exhibit sharp streaks extending along the stacking direction, while those of the family reflections appear as discrete spots. Figures S2(a-d) schematically illustrate electron diffraction patterns calculated for the OD-groupoid family formed with the C_1 stacking positions (Figs. S2(a, c)) and for those formed with either C_2 or C_3 stacking positions (Figs. S2(b, d)) for the incident beam directions corresponding to $[2\bar{1}\bar{1}0]$ and $[1\bar{1}00]$ of Mg with the hcp structure. For the OD-groupoid family formed with the C_1 stacking positions, family reflections appear in the $n/2[01\bar{1}l]^*$ and $n/2[11\bar{2}l]^*$ reciprocal lattice rows (n: all integer) in the $[2\bar{1}\bar{1}0]$ and $[1\bar{1}00]$ SAED patterns, respectively[1,2]. However, characteristic reflections appears in the $n/2[01\bar{1}l]^*$ and $n/2[11\bar{2}l]^*$ reciprocal lattice rows (n: odd integer) for the OD-groupoid family formed with either C_2 or C_3 stacking positions because their family structure has a smaller in-plane unit cell ($\sqrt{3}a_{\mathrm{Mg}} \times \sqrt{3}a_{\mathrm{Mg}}$). If stacking positions other than C_1 (i.e., 'non-equivalent stacking' described with positions C_2 and C_3 in Fig. S1(b)) are incorporated as minor inclusions, discrete spots of family reflections for the OD-groupoid family formed with the C_1 stacking positions are accompanied by weak streaks in the $n/2[01\bar{1}l]^*$ and $n/2[11\bar{2}l]^*$ reciprocal lattice rows (n: odd integer).

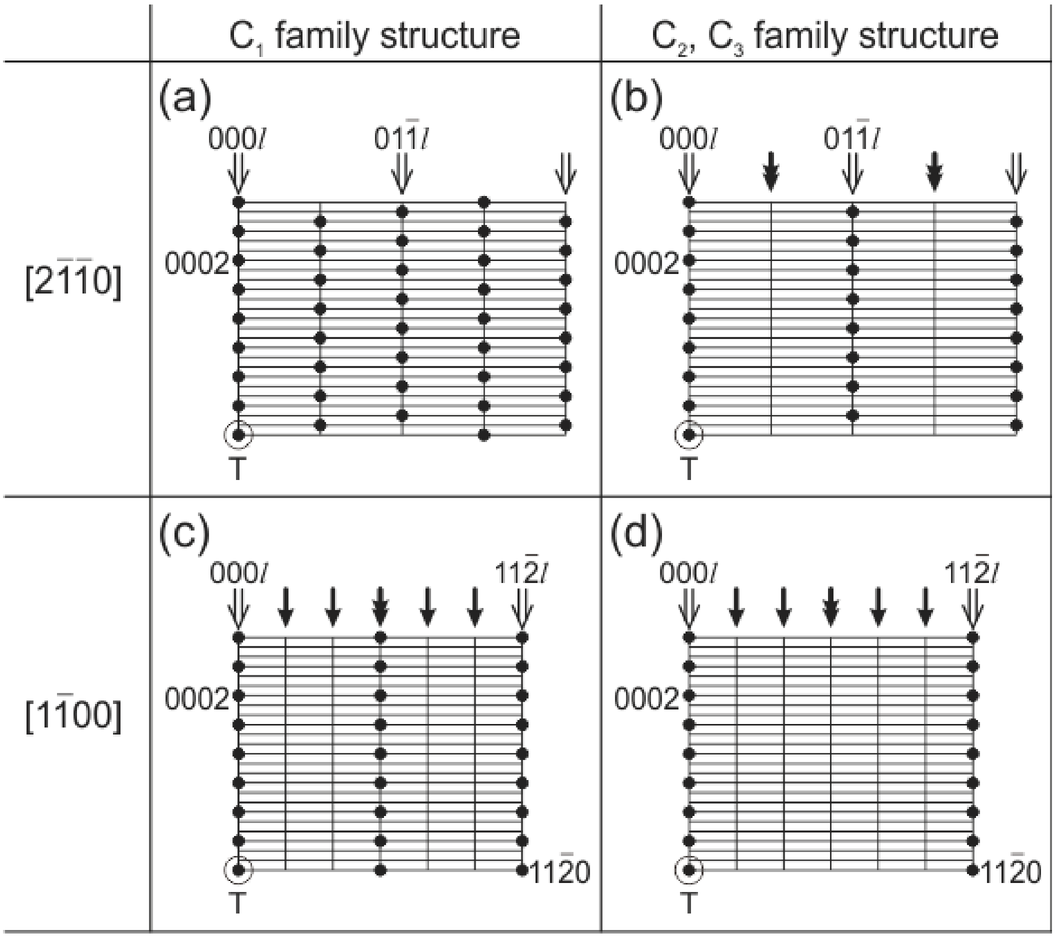

Figure S2. Schematic SAED patterns for the family structures for the 18*R*-LPSO-type Mg-Al-Gd OD phase in the $[2\bar{1}\bar{1}0]$ and $[1\bar{1}00]$ projections. (a,c) C_1 and (b,d) C_2 and C_3 family structures. Double arrows indicate the reciprocal lattice rows of the family reflections common to the C_1, C_2 and C_3 family structures. Two-headed arrows indicate the reciprocal lattice rows of the family reflections for the C_1 family structure and the characteristic reflections for the C_2 and C_3 family structures. One-headed arrows indicates the reciprocal lattice rows of the characteristic reflections for all family structures.

SAED patterns of a precipitate of the OD phase in the Mg-Al-RE (RE = Y, Dy, Ho, Er, Nd and Sm) ternary systems taken along $[2\bar{1}\bar{1}0]$ and $[1\bar{1}00]$ directions are shown in Fig. S3. The observations were made after annealing at 450 °C for 64 h (Figs. S3(a, c, d, e, g, h, i, l)) and 525 °C for 64 h (Figs. S3(b,f)) for the OD phases in the Mg-Al-RE (RE = Y, Dy, Ho, Er) systems, while for the OD phase in the Mg-Al-RE (RE = Nd and Sm) systems, the as-solidified specimens were used for the observations (Figs. S3(j, k, m, n)). In all SAED patterns, superlattice spots are observed in the $[000l]^*$ systematic row at positions dividing the distance between the transmitted beam and $[0002]^*$ fundamental reflection (corresponding to the interplanar spacing of close-packed planes) by six, indicating that the number of close-packed atomic planes comprising each of structural blocks is mostly six. The existence of discrete diffraction spots in the $n/2[01\bar{1}l]^*$ and $n/2[11\bar{2}l]^*$ reciprocal lattice rows (n: odd integer) and intense streaks in the $n/6[11\bar{2}l]^*$ reciprocal lattice rows (n = 1, 2, 4, and 5) in the SAED patterns indicates that the OD phase in these six alloy systems mostly have OD structures formed by preferentially stacking 6-layer structural blocks with the C_1-type stacking relations as previously observed in the Mg-Al-Gd system[1]. The existence of weak streaks extending along the $n/2[01\bar{1}l]^*$ and $n/2[11\bar{2}l]^*$ reciprocal lattice rows (n: odd integer) indicates slight incorporation of the C_2- or C_3-types stacking relations in the OD phases in the Mg-Al-RE (RE = Y, Dy, Ho and Er) systems (groups 1 and 3). In addition, the diffuse nature appearing as sharp and intense streaks in the reciprocal lattice rows of the characteristic reflections in the $[1\bar{1}00]$ SAED patterns indicates the one-dimensionally disordered nature after heat treatment at 450 °C for 64 h. When the annealing

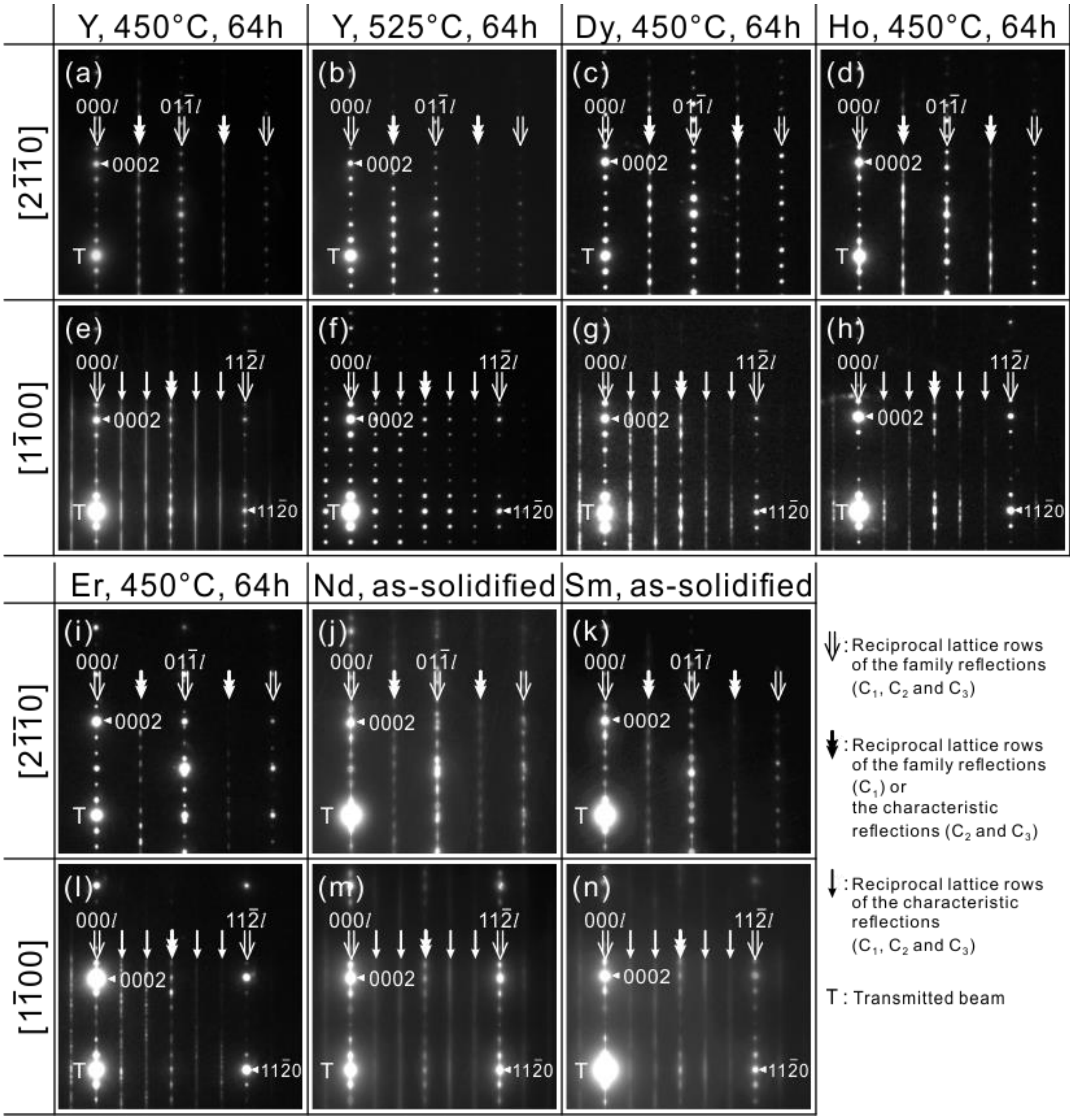

Figure S3. Experimental SAED patterns of the Mg-Al-RE OD phase in (a,c,d,e,g,h,i,l) the ingots heat-treated at 450 °C for 64 hours, (b,f) the ingots heat-treated at 525°C for 64 hours and (j,k,m,n) the as-solidified ingots taken along (a-d, i-k) $[2\bar{1}\bar{1}0]$ and (e-h, l-n) $[1\bar{1}00]$. RE = (a,b,e,f) Y, (c,g) Dy, (d,h) Ho, (i,l) Er, (j,m) Nd and (k,n) Sm.

temperature is increased to 525 °C (Figs. S3(b, f)), all of the streaks in the $[1\bar{1}00]$ SAED pattern for the OD phase in the Mg-Al-Y system is eliminated in most of relatively thick precipitates, indicating the progress in the stacking ordering of these structural blocks in addition to the in-plane ordering of Al_6Y_8 atomic clusters in the structural block. Actually, the SAED patterns of the OD phase in the Mg-Al-Y system (Figs. S3(b, f)) are indexed consistently as those of the MDO polytype, 1*M* (MDO1, space group: *C*2/*m*) with the simplest stacking of the structural blocks in the OD-groupoid family formed with the C_1-type stacking relations as observed in the Mg-Al-Gd OD phase[8]. In contrast to the Mg-Al-RE OD phase in the groups 1 and 3 ternary systems, the SAED patterns of the as-cast specimens of the Mg-Al-Nd and Mg-Al-Sm ternary systems (group 2) exhibit somewhat diffuse nature of all diffraction spots. This suggests the incomplete in-plane ordering of Al_6RE_8 clusters as well as the coexistence of different structural blocks composed of different numbers of atomic layers, which is further confirmed by the atomic resolution HAADF-STEM observations described in the main text.

S3. First-principles calculations

Table S1 summarizes the OD-phase formation energy ΔE_{form}, stability factor ΔE_{stab} and energy required to insert one additional atom *i* (*i* = Mg, Al or RE) in each Al_6RE_8 atomic cluster ΔE_{ins} (insertion energy) evaluated with equations (1) – (3) in the main text. Each convex hull is composed of three convex hull phases as listed in Table 1 mostly selected according to the selections made by Saal and Wolverton except for the Mg-Al-Pm ternary system. For the Mg-Al-Pm systems, Mg (hcp), $Mg_{41}Pm_5$ ($Mg_{41}Ce_5$-type) and Al_2Pm (Cu_2Mg-type) were referred to as convex hull phases instead of Mg(hcp), Mg_3Pm ($D0_{22}$) and Al_3Pm ($D0_{19}$) used by Saal and Wolverton[10], since it would be more reasonable to assume that the selection of convex hull phases is identical to those for the Mg-Al-RE ternary systems with RE = Nd and Sm, which are neighboring elements of Pm. With the different selection of the convex hull phases, the ΔE_{stab} value for the Mg-Al-Pm OD phase (-0.72 meV/atom) is reasonably located between those for the Mg-Al-Nd and Mg-Al-Sm OD phases (2.61 and -3.21 meV/atom, respectively), which differs from the anomalously low value of -13 meV/atom reported in ref. 10.

References

[1] Yokobayashi, H. *et al.* Enrichment of Gd and Al atoms in the quadruple close packed planes and their in-plane long-range ordering in the long period stacking-ordered phase in the Mg-Al-Gd system. *Acta Mater.* **59**, 7287-7299 (2011).

[2] Kishida, K., Yokobayashi, H., Inoue, A. & Inui, H. Crystal Structures of Long-Period Stacking-Ordered Phases in the Mg-TM-RE Ternary Systems. *MRS Symp. Proc.* **1516**, 291-302 (2013).

[3] Dornberger-Schiff, K. *Abh. Dtsch. Akad. Wiss. Berlin, Kl Chem. Geol. Biol.* **3**, 1-107 (1964).

[4] *International Table for Crystallography, 2nd ed. Vol. E* (eds. Kopsky, V. & Litvin, D. B.) (Wiley, 2010).

[5] Ferraris, G., Makovicky, E. & Merlino, S. *Crystallography of Modular Materials.* (Oxford University Press, 2004).

[6] Okamoto, N.L., Yasuhara, A. & Inui, H. Order-disorder structure of the δ_{1k} phase in the Fe-Zn system determined by scanning transmission electron microscopy. *Acta Mater.* **81**, 345-357 (2014).

[7] Iwatake, Y. *et al.* New crystal structure of Nd_2Ni_7 formed on the basis of stacking of block layers. *Int. J. Hydrogen Energy* **40**, 3023-3034 (2015).

[8] Kishida, K., Yokobayashi, H. & Inui, H. The most stable crystal structure and the formation processes of an order-disorder (OD) intermetallic phase in the Mg-Al-Gd ternary system. *Philos. Mag.* **93**, 2826-2846 (2013).

[9] Kishida, K. *et al.* The crystal structure of the LPSO phase of the 14*H*-type in the Mg-Al-Gd alloy system. *Intermetallics* **31**, 55-64 (2012).

[10] Saal, J.E. & Wolverton, C. Thermodynamic stability of Mg-based ternary long-period stacking ordered structures. *Acta Mater.* **68**, 325-338 (2014).

Table S1. Formation energy ΔE_{form}, stability factor ΔE_{stab} and insertion energy ΔE_{ins} for the 18*R*-LPSO-type Mg-Al-RE OD phase.

RE	Formation energy ΔE_{form} (meV/atom)				Stability factor ΔE_{stab} (meV/atom)				Insertion energy ΔE_{ins} (eV/incl.)			Convex hull phases
	No incl.	+Mg	+Al	+RE	No incl.	+Mg	+Al	+RE	+Mg	+Al	+RE	
Y	-75.2	-101.2	-100.3	-103.4	18.8	-8.52	1.20	-5.74	-1.98	-1.91	-2.14	Mg, MgAlY, Mg_3Y
La	-67.2	-91.0	-88.3	-88.9	47.1	22.0	30.0	30.2	-1.81	-1.60	-1.65	$Mg_{12}La$, Al_2La, Mg_3La
Ce	-69.7	-95.2	-92.5	-92.2	39.4	12.5	20.2	23.7	-1.93	-1.73	-1.71	$Mg_{12}Ce$, Al_2Ce, $Mg_{41}Ce_5$
Pr	-71.2	-97.8	-95.5	-95.1	35.0	7.01	15.0	17.9	-2.02	-1.85	-1.82	$Mg_{12}Pr$, Al_2Pr, $Mg_{41}Pr_5$
Nd	-71.8	-99.1	-97.3	-96.9	31.3	2.61	10.8	12.8	-2.07	-1.93	-1.90	Mg, Al_2Nd, $Mg_{41}Nd_5$
Pm	-72.5	-100.3	-98.8	-98.7	28.5	-0.72	7.69	8.25	-2.11	-2.00	-1.98	Mg, Al_2Pm, $Mg_{41}Pm_5$
Sm	-71.2	-99.2	-97.9	-98.1	26.2	-3.21	5.42	4.39	-2.12	-2.03	-2.04	Mg, Al_2Sm, $Mg_{41}Sm_5$
Eu	-33.8	-49.4	-46.3	-51.2	40.9	24.3	32.9	27.6	-1.17	-0.95	-1.31	Mg, Al_2Eu, Mg_2Eu
Gd	-71.3	-99.6	-98.7	-99.9	21.7	-7.85	1.18	-2.93	-21.4	-2.08	-2.16	Mg, Al_2Gd, Mg_3Gd
Tb	-70.5	-97.2	-96.5	-98.1	19.6	-8.31	0.81	-4.50	-2.02	-1.97	-2.09	Mg, Al_2Tb, Mg_3Tb
Dy	-68.4	-94.2	-93.7	-95.7	18.4	-8.61	0.57	-5.92	-1.95	-1.92	-2.07	Mg, Al_2Dy, Mg_3Dy
Ho	-65.7	-90.8	-90.3	-92.8	17.5	-8.66	0.57	-7.07	-1.89	-1.86	-2.04	Mg, Al_2Ho, Mg_3Ho
Er	-62.7	-87.0	-86.7	-89.6	17.7	-7.65	1.54	-6.97	-1.83	-1.81	-2.02	Mg, Al_2Er, $Mg_{24}Er_5$
Tm	-58.8	-82.2	-82.0	-85.3	16.5	-7.96	1.34	-8.64	-1.77	-1.75	-2.00	Mg, Al_2Tm, Mg_3Tm
Yb	-21.2	-41.3	-38.7	-43.7	42.4	21.5	29.2	23.1	-1.48	-1.30	-1.66	Mg, Al_2Yb, Mg_2Yb
Lu	-52.3	-74.3	-74.2	-78.3	19.9	-3.09	5.81	-4.83	-1.66	-1.65	-1.95	Mg, Al_2Lu, $Mg_{24}Lu_5$
